# Arthroscopy for Femoroacetabular Impingement in Athletes Versus Non-Athletes: Systematic Review and Meta-Analysis

**DOI:** 10.3390/healthcare13050470

**Published:** 2025-02-21

**Authors:** Filippo Migliorini, Nicola Maffulli, Tommaso Bardazzi, Swaminathan Ramasubramanian, Naveen Jeyaraman, Madhan Jeyaraman

**Affiliations:** 1 Department of Life Sciences, Health, and Health Professions, Link Campus University of Rome, 00165 Rome, Italy; 2 Department of Orthopaedic and Trauma Surgery, Academic Hospital of Bolzano (SABES-ASDAA), 39100 Bolzano, Italy; tommaso.bardazzi@sabes.it; 3 Department of Trauma and Orthopaedic Surgery, Faculty of Medicine and Psychology, University La Sapienza, 00185 Rome, Italy; nicola.maffulli@uniroma1.it; 4 School of Pharmacy and Bioengineering, Faculty of Medicine, Keele University, Stoke on Trent ST4 7QB, UK; 5 Centre for Sports and Exercise Medicine, Barts and the London School of Medicine and Dentistry, Mile End Hospital, Queen Mary University of London, London E1 4DG, UK; 6 Department of Internal Medicine, Government Medical College, Omandurar Government Estate, Chennai 600018, Tamil Nadu, India; swaminathan.ramasubramanian@outlook.com; 7 Department of Orthopaedics, ACS Medical College and Hospital, Dr. MGR Educational and Research Institute, Chennai 600077, Tamil Nadu, India; naveenjeyaraman@yahoo.com (N.J.); madhanjeyaraman@gmail.com (M.J.)

**Keywords:** femoroacetabular impingement, hip, pain, sports, athletes

## Abstract

Background: Femoroacetabular impingement (FAI) is a frequently observed hip condition among young, active individuals—especially athletes—that can result in pain, restricted mobility, and a heightened risk of osteoarthritis. Hip arthroscopy has increasingly become the preferred surgical approach for managing FAI due to its ability to alleviate symptoms and improve function. However, potential differences in outcomes between athletes and non-athletes have not been thoroughly investigated. This systematic review and meta-analysis compared arthroscopic management for FAI in athletes versus non-athletes. The outcomes of interest were patient-reported outcome measures (PROMs) and complications. Methods: PubMed, Web of Science, and Embase were systematically accessed until October 2024. The studies eligible were clinical investigations comparing athletes and non-athletes undergoing hip arthroscopy for FAI with a minimum follow-up of 24 months. The outcomes assessed included the Visual Analogue Scale (VAS), Hip Outcome Score for Activities of Daily Living (HOS-ADL), and the Hip Outcome Score–Sport-Specific Subscale (HOS-SSS). Data on reoperation rates and progression to total hip arthroplasty were also extracted. The ROBINS-I tool was used to assess the risk of bias, and meta-analyses were performed using Review Manager 5.3. Results: Three comparative investigations, comprising 808 patients (165 athletes and 643 non-athletes), met the inclusion criteria. Baseline characteristics were similar across both groups. The analyses demonstrated no statistically significant differences in the PROMs (VAS: *p* = 0.7; HOS-ADL: *p* = 0.5; HOS-SSS: *p* = 0.4), reoperation rates (*p* = 0.7), or the rate of progression to arthroplasty (*p* = 0.4) between athletes and non-athletes. Furthermore, meta-analyses of two studies reinforced the absence of significant differences in VAS and HOS-SSS outcomes. Conclusion: Hip arthroscopy for FAI appears to yield equivalent improvements in pain and functional outcomes for both athletes and non-athletes, with comparable complication rates at an approximate two-year follow-up. Despite the limited number of studies and a moderate risk of bias, the findings support the effectiveness of arthroscopic intervention across varying physical activity levels.

## 1. Introduction

Femoroacetabular impingement (FAI) is characterised by abnormal contact between the femoral head and the acetabulum [1,2]. The articular incongruence in FAI leads to labral and cartilage damage [3,4,5]. If this continuous and repetitive damage is left untreated, FAI also increases the risk of developing osteoarthritis (OA) and the need for total hip arthroplasty (THA) [6,7,8]. Two main types of FAI are described: cam impingement and pincer impingement [9,10,11,12]. A mixed-type morphology, including cam and pincer characteristics, is also reported and is the most frequent form [13,14,15]. Cam deformity is characterised by a not perfectly round femoral head with a bony prominence at the junction of the femoral head and neck, located mainly at the anterosuperior aspect of the neck of the femur [16,17]. Pincer impingement consists of over-coverage of the femoral head by the acetabulum and is the most common form of FAI in women [18,19,20,21]. While cam impingement occurs more frequently in men and athletes, pincer impingement has a higher incidence in women and is less commonly found in athletes [6,9,22]. A mixed-type morphology, including pincer and cam types, is also reported and is the most frequent form [17,23]. FAI predominantly affects young and active individuals, including a significant number of athletes who experience hip pain and a limited range of motion due to repetitive, high-demand physical activities [24,25]. The increased prevalence of FAI in athletes is well documented and mainly involves sports requiring extreme hip movements, such as soccer and hockey [26,27]. Several management strategies, including non-operative care (e.g., physical therapy, activity modifications, injection therapy) and surgical interventions, have been developed for FAI syndrome [28,29,30]. In recent decades, hip arthroscopy has emerged as the preferred surgical intervention for FAI, offering the benefits of minimally invasive techniques, faster recovery times, and reduced post-operative pain [31,32,33,34,35]. The procedure involves reshaping the femoral head and acetabulum to alleviate impingement and repair any associated chondrolabral tears [28,36,37,38]. Research has demonstrated the effectiveness of hip arthroscopy in improving clinical outcomes [39,40,41], particularly in athletes motivated to return to their pre-injury level of sport participation [42,43,44]. A ten-year follow-up study highlighted the positive long-term outcomes and high rates of return to sport among athletes who underwent hip arthroscopy for FAI [45]. Despite the growing body of literature on hip arthroscopy for FAI, there still is a significant gap in understanding the difference in outcomes between athletes and non-athletes [31,46].

The current evidence often focuses on short-term outcomes, highlighting the need for long-term outcomes. Additionally, comprehensive analyses lack a comparison of the recovery trajectories and functional outcomes of athletes versus non-athletes following arthroscopic intervention for FAI. For instance, while competitive athletes generally report superior outcomes after surgery, the specific factors contributing to these differences require further exploration [47]. Variations in rehabilitation protocols and their impact on recovery and return-to-sport timelines between these groups are poorly documented. Furthermore, quicker return-to-sport times are expected in professional athletes, although the underlying reasons, whether due to physiological differences, better rehabilitation programs, or other factors, remain unclear [48]. There is also a need for more detailed investigations into the biomechanical and anatomical differences that may influence surgical outcomes. Previous research has highlighted the importance of anatomical considerations in surgical planning and outcome prediction, but these findings are often not specific to athletic versus non-athletic populations [49,50,51]. Moreover, the psychological aspects of returning to sport have not been thoroughly examined for their particular impact on FAI when comparing athletes versus non-athletes. Understanding these nuances can help tailor post-operative care and rehabilitation strategies to better meet the expectations of both groups. This systematic review and meta-analysis evaluated the outcomes of arthroscopic treatment for FAI in athletes compared to non-athletes, focusing on patient-reported outcome measures (PROMs) and the incidence of reoperation and the progression to THA.

## 2. Materials and Methods

### 2.1. Eligibility Criteria

All comparative investigations addressing the arthroscopic management of FAI were included for consideration. Only clinical investigations comparing two distinct groups based on sport activity levels were suitable (athletes versus non-athletes). Athletes were defined as individuals engaged in competitive or high-level sport activities. Non-athletes were defined as individuals with recreational or non-competitive activity levels. Eligible studies must specify the type of sports involved, encompassing individual and team sports, such as soccer, hockey, basketball, and track and field. Eligible studies were required to be published in peer-reviewed journals in one of the following languages, reflecting the linguistic expertise of the authors: English, Spanish, German, French, or Italian. Inclusion was restricted to studies classified as Level I to III evidence according to the 2020 guidelines of the Oxford Centre for Evidence-Based Medicine [52]. Editorials, letters to the editor, opinion articles, and review papers were excluded. Furthermore, studies involving in vitro models, animal subjects, biomechanical evaluations, computational modelling, or cadaveric research were deemed ineligible. Only studies with a minimum of 24 months of follow-up were considered to ensure the assessment of long-term outcomes and durability of arthroscopic interventions.

### 2.2. Search Strategy

This systematic review followed the 2020 Preferred Reporting Items for Systematic Reviews and Meta-Analyses (PRISMA) guidelines [53]. The literature search was organised using the following framework:

- P (Problem): femoroacetabular impingement (FAI);
- I (Intervention): arthroscopy;
- C (Comparison): athletes versus non-athletes;
- O (Outcomes): patient-reported outcome measures (PROMs) and complications;
- T (Timings): A minimum follow-up period of 24 months;
- D (Design): clinical study.

In October 2024, PubMed, Web of Science, and Embase were accessed without additional filters or temporal restrictions. The Medical Subject Headings (MeSHs) used during the search are detailed in the Supplementary Materials.

### 2.3. Selection and Data Collection

Two authors (F.M. and T.B.) manually screened the titles retrieved through the search and reviewed relevant abstracts. Subsequently, full-text articles were assessed to ensure compliance with the pre-established inclusion criteria. Studies lacking accessible full-text versions were excluded from the analysis. Furthermore, the reference lists of included articles were systematically reviewed to identify additional studies that met the eligibility requirements. Discrepancies between the authors were resolved through arbitration by a senior investigator (N.M.), who provided the final adjudication.

### 2.4. Data Items

Extracted information included general study characteristics, namely, the surname of the primary author, publication year, journal, and length of the follow-up. Baseline characteristics collected included the total number of enrolled patients, the proportion of female participants, and body mass index (BMI). Data concerning the Visual Analogue Scale (VAS) [54], Hip Outcome Score-Activities of Daily Living (HOS-ADL) and Hip Outcome Score-Sport-Specific Subscale (HOS-SSS) [55] were collected at baseline and at the last follow-up. Data concerning hip arthroscopy revision surgeries and progression to THA were also collected. The VAS is a widely used tool to measure subjective pain intensity. It consists of a continuous scale (typically 10 cm or 100 mm) where patients mark their pain level, ranging from “no pain” (0) to “worst pain imaginable” (10 or 100). Its simplicity and ease of use make it a standard in clinical settings and research for evaluating pain outcomes. The HOS-ADL is a PROM designed to assess hip-related function during everyday activities. It includes a series of questions that evaluate a patient’s ability to perform tasks such as walking, sitting, or climbing stairs, with responses scored on a Likert scale. Higher scores indicate better functional capacity in daily life. The HOS-SSS is a complementary subscale to the HOS-ADL that focuses on assessing hip function during sport-specific activities. It includes questions targeting high-demand tasks, such as running, jumping, and pivoting, which are particularly relevant to active individuals and athletes. Like the HOS-ADL, higher scores indicate better performance and fewer limitations. Extraction was performed using Microsoft Office Excel version 16.0 (Microsoft Corporation, Redmond, WA, USA).

### 2.5. Assessment of the Risk of Bias

Two authors (F.M. and T.B.) assessed the risk of bias following the Cochrane Handbook for Systematic Reviews of Interventions [56] guidelines. The Risk of Bias in Nonrandomized Studies of Interventions (ROBINS-I) tool was used [57]. The ROBINS-I tool systematically evaluates the potential risk of bias across seven predefined domains: confounding factors, patient selection characteristics before intervention, bias in the classification of interventions, deviations from intended interventions, incomplete outcome data, inaccuracies in outcome measurement, and selective reporting of outcomes. The results of the ROBINS-I assessments were graphically represented using the RobVis software (Risk-of-Bias VISualization, Riskofbias.info, Bristol, UK) [58].

### 2.6. Synthesis Method

Statistical analyses were performed following the Cochrane Handbook for Systematic Reviews of Interventions [56], using IBM SPSS software version 25 (International Business Machines Corporation, Armonk, NY, USA). The mean difference and standard deviation were calculated for descriptive statistics. Baseline comparability was evaluated using the *t*-test, with *p* > 0.05 indicating satisfactory comparability. Continuous data were reported using arithmetic means and standard deviations, while dichotomous variables were presented as frequencies (events/observations). Meta-analyses were conducted with Review Manager version 5.3 (The Nordic Cochrane Collaboration, Copenhagen, Denmark). The inverse variance method was applied for continuous data using mean difference (MD) as the effect measure, with a 95% confidence interval (CI). Heterogeneity was assessed using the Higgins-I^2^ and χ^2^ tests. A P_χ2_ > 0.05 indicates no statistically significant heterogeneity, whereas a P_χ2_ < 0.05 necessitates further evaluation of heterogeneity based on Higgins-I^2^ values: low (<30%), moderate (30–60%), or high (>60%). A fixed-effect model was used as the default approach; a random-effect model was applied in cases of substantial heterogeneity. Statistical significance was set at *p* < 0.05.

## 3. Results

### 3.1. Study Selection

**Figure 1:**
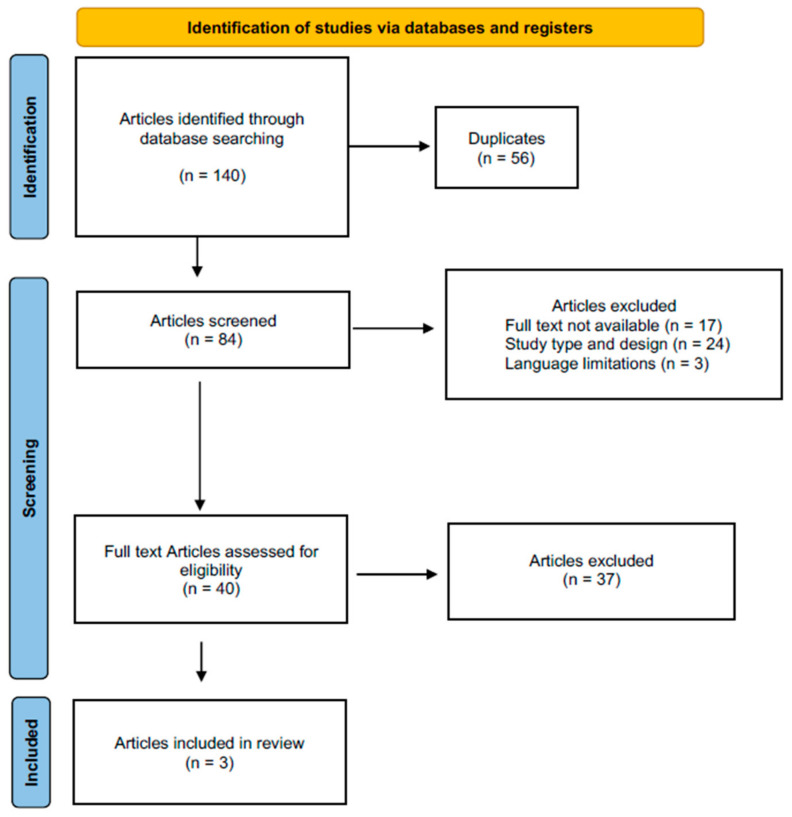
PRISMA flow chart of the literature search.

A total of 140 comparative studies were found. After duplicates were removed, the abstracts of 84 articles were screened for eligibility. A total of 37 articles were excluded for various reasons. These included incompatible study design criteria (n = 24), no full-text availability (n = 17), and language barriers (n = 3). Another 37 articles were excluded after a full-text review of the remaining 40. Consequently, a final selection of three studies was included in this systematic review. All studies included are retrospective in design. The results of the literature search are shown in Figure 1.

### 3.2. Risk of Bias Assessment

**Figure 2:**
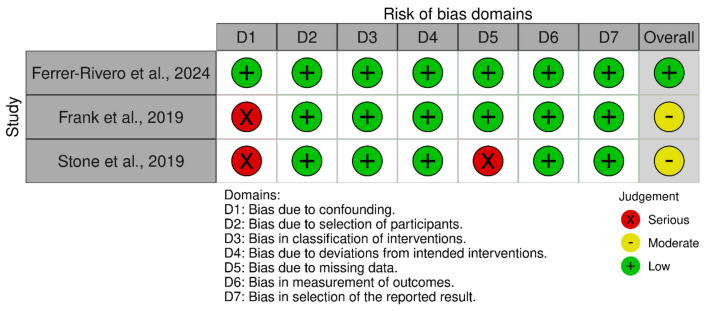
The ROBINS-I of non-RCTs.

The Risk of Bias in Nonrandomized Studies of Interventions (ROBINS-I) tool was employed to evaluate the risk of bias in the three non-randomised studies included. A significant concern was identified in the domain of confounding, with two out of the three studies demonstrating a serious risk of bias due to confounding factors. This represents a notable limitation in the methodological quality of these studies. In contrast, the risk of bias associated with participant selection and intervention classification was uniformly low across all studies, and no deviations from the intended intervention protocols were observed. However, one study raised concerns related to missing outcome data. The domains of outcome measurement and selective reporting of results were consistently free from bias in all three studies. Overall, the ROBINS-I assessment indicated a moderate risk of bias in two studies and a low risk of bias in the third study. While the methodological quality of the included studies is generally acceptable, the potential for confounding bias in two studies requires careful consideration (Figure 2).

### 3.3. Study Characteristics and Results of Individual Studies

Data from 808 patients were collected. The mean length of follow-up was 26.1 ± 3.3 months. The mean age was 33.1 ± 2.5 years, and the mean BMI was 24.8 ± 1.1 kg/m^2^. An overview of the included studies is shown in Table 1.

### 3.4. Baseline Comparability

Comparability was found at baseline in the mean length of follow-up, mean age, mean BMI, women, VAS, HOS-ADL, and HOS-SSS (Table 2).

### 3.5. Synthesis of Results

No significant difference was found in VAS, HOS-ADL and HOS-SSS between the two cohorts (Table 3).

No statistically significant difference in the rate of reoperation (*p* = 0.7) and progression to THA (*p* = 0.4) was found (Table 4).

### 3.6. Meta-Analyses

**Figure 3:**
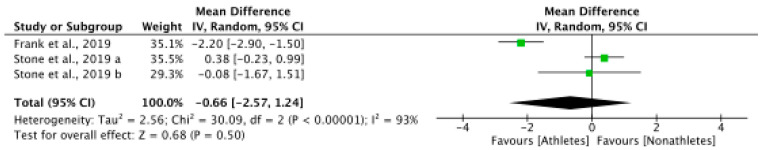
Forest plots of this meta-analysis: VAS.

Two comparative studies reported data on the VAS and were included in this meta-analysis [60,61]. The final effect evidenced no differences in VAS (*p* = 0.5) (Figure 3).

**Figure 4:**
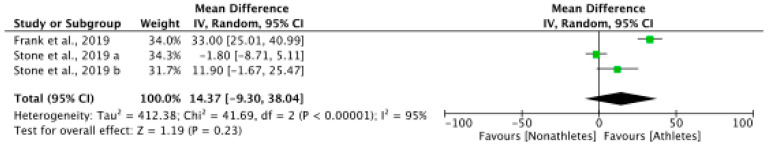
Forest plots of this meta-analysis: HOS-SSS.

Two comparative studies reported data on the HOS-SSS and were included in this meta-analysis [60,61]. The final effect evidenced no differences in HOS-SSS (*p* = 0.2) (Figure 4).

## 4. Discussion

This systematic review and meta-analysis comparing the outcomes of hip arthroscopy for FAI between athletes and non-athletes reveal valuable insights into the effectiveness of this intervention across different patient populations. The methodological quality of the included studies, assessed through the ROBINS-I tool, indicated a moderate risk of bias in two studies and a low risk of bias in one study. Despite the limitations and the retrospective nature of the studies [59,60,61], a consistent improvement in PROMs across the whole population suggests that hip arthroscopy is a reliable intervention for FAI.

The baseline comparability between athlete and non-athlete groups was maintained regarding mean follow-up duration, mean age, mean BMI, and gender distribution. This strengthens the validity of the comparative analysis (Table 2). The exclusion of studies based on full-text availability and language barriers might have introduced selection bias, although the impact of this bias on the overall findings is unclear. The meta-analysis of the results demonstrated no significant differences in VAS, HOS-ADL, and HOS-SSS between athletes and non-athletes (Table 3). The lack of significant differences in PROMs between groups suggests that athletes and non-athletes can expect similar improvements in functional outcomes and pain relief following hip arthroscopy. This outcome similarity may be attributed to the effective surgical techniques and standardised rehabilitation protocols employed across both populations. Additionally, both groups likely adhere to post-operative guidelines rigorously, contributing to comparable recovery trajectories. No significant difference between groups was observed in reoperation and progression to total hip arthroplasty (THA). However, athletes’ physical demands and higher performance expectations may necessitate more intensive or specialised rehabilitation to achieve optimal functional recovery, which was not uniformly addressed in the included studies. Despite no significant difference, the higher trend of return to sports in the non-athlete group compared to athletes warrants further investigation (Table 4). Athletes often have higher baseline expectations and more demanding physical activity levels, which might influence their perception of recovery and RTS rates [59]. Psychological factors such as motivation, fear of re-injury, and pressure to return to sport may differentially impact athletes, potentially masking differences in physical recovery outcomes. This disparity highlights the need for tailored rehabilitation programs that address the specific demands and expectations of athletes undergoing hip arthroscopy.

Ferrer-Rivero et al. compared the outcomes of 11 high-level female athletes with 22 non-athlete females after hip arthroscopy for FAI [59]. The mean follow-up period was 32.4 months for athletes and 31.2 months for non-athletes [59]. The primary outcomes assessed were PROMs such as the Non-Arthritic Hip Score (NAHS), International Hip Outcome Tool (iHOT-33), Hip Outcome Score-Daily Living Activities (HOS-DLA), and Hip Outcome Score-Sport Specific Subscale (HOS-SSS) [59]. Both groups significantly improved scores from preoperative to the last follow-up visit. For example, the NAHS improved from a median of 67 to 93.3 in athletes and from 57.8 to 85 in non-athletes. Similarly, the HOS-SSS improved from 51.4 to 75 in athletes and 52.7 to 83 in non-athletes. Despite the improvements, the study found no significant differences between athletes and non-athletes regarding the final PROMs. Hip arthroscopy is equally effective in improving clinical outcomes in high-level females and non-athletes. The lower rate of return to sports (RTS) in athletes (63.6%) compared to non-athletes (85.7%) was noted, but this difference was not statistically significant. The authors concluded that hip arthroscopy is a beneficial intervention for FAI in high-level female athletes, with no evidence of a ceiling effect in sport-related outcomes.

Frank et al. conducted a comprehensive retrospective analysis involving 194 female patients, grouped into 97 athletes and 97 non-athletes (97 in each group) [60]. The mean follow-up duration was 31.2 months for both groups. The study primarily focused on PROMs such as the Visual Analogue Scale (VAS) for pain, HOS-ADL, and HOS-SSS. The results showed significant improvements in all PROMs for both groups. For athletes, the VAS score improved from 6.8 to 1.5, HOS-ADL from 66.9 to 91.6, and HOS-SSS from 43.2 to 82.5. Similarly, non-athletes showed improvements, with VAS scores reducing from 6.7 to 2.1, HOS-ADL increasing from 55.3 to 74.6, and HOS-SSS from 42.1 to 73.2. No significant differences were found between athletes and non-athletes in the outcomes, indicating that hip arthroscopy is equally effective in both groups. The study also evaluated the rate of complications, including reoperation and progression to total hip arthroplasty (THA). Both groups had low rates of complications, with no significant differences observed. Hence, hip arthroscopy provides substantial clinical benefits for patients with FAI, regardless of their athletic status.

Stone et al. [61] conducted the largest study of the three, including 464 non-athletes and 47 athletes. The mean follow-up period for non-athletes was 24 months, and it was slightly shorter for athletes. This study focused on PROMs such as the VAS for pain, HOS-ADL, and HOS-SSS. Similar to the other studies, they found significant improvements in all PROMs for athletes and non-athletes [61]. For instance, the VAS score improved from 6.8 to 1.5 in athletes and 6.7 to 2.1 in non-athletes [61]. HOS-ADL improved from 66.9 to 91.6 in athletes and 55.3 to 74.6 in non-athletes [61]. HOS-SSS scores increased from 43.2 to 82.5 in athletes and 42.1 to 73.2 in non-athletes [61]. The study also highlighted the baseline comparability between the two groups regarding demographic characteristics and preoperative PROMs, strengthening the validity of the comparative analysis [61]. The low rates of reoperations and THA progression, as well as the similar improvements in PROMs between athletes and non-athletes, further emphasise the effectiveness of hip arthroscopy in treating FAI.

One of the strengths of this systematic review is the focus on a specific and clinically relevant question, namely, the comparative effectiveness of hip arthroscopy in athletes versus non-athletes. Including a homogeneous patient population with well-defined criteria enhances the clinical applicability of the findings. Additionally, using validated PROMs, specifically designed for young active patients with hip disorders, provides a comprehensive assessment of patient outcomes, capturing both functional improvement and pain relief. The clinical implications of these findings suggest that hip arthroscopy can be confidently recommended for both athletes and non-athletes suffering from FAI, potentially leading to improved quality of life and sustained physical activity levels. However, several limitations must be acknowledged. The small number of included studies limits the generalisability of the findings and the statistical power of the meta-analyses. The moderate risk of bias in two studies, particularly related to confounding, underscores the need for high-quality, prospective RCTs to confirm these results. The variability in rehabilitation protocols across studies may affect the consistency of patient outcomes, indicating a need for standardised post-operative care protocols. Furthermore, the heterogeneity in follow-up durations, the potential for selection bias due to exclusion criteria and the arbitrary definition of athletes might have influenced the outcomes. Despite the limited number of included studies (n = 3), the findings emphasise the overall efficacy of hip arthroscopy in alleviating symptoms and enhancing patient-reported outcome measures (PROMs) in both athletes and non-athletes. A review of the 37 excluded studies revealed that many did not meet the predefined inclusion criteria, primarily due to mismatches in study design, lack of full-text availability, and language barriers. Of these excluded studies, several were prospective cohorts or randomised controlled trials that could provide higher levels of evidence but were excluded based on the selection criteria. Others focused on different populations or used different surgical techniques that were not pertinent to the current analysis. This exclusion highlights the stringent criteria applied to ensure the relevance and quality of the included studies. However, it also underscores the limited availability of high-quality research comparing athletes and non-athletes undergoing hip arthroscopy for FAI. To ensure a comprehensive and unbiased literature search, we followed established guidelines for conducting systematic reviews, such as the PRISMA (Preferred Reporting Items for Systematic Reviews and Meta-Analyses) guidelines. While no universally mandated number of databases is required for a systematic review, the selection of databases should be guided by their relevance to the topic under investigation. For this investigation, we carefully accessed Web of Science, PubMed, and Embase, which are recognised as leading medical and sport medicine sources. These databases collectively cover a wide range of relevant and high-quality literature on our topic. No other sources (e.g., grey literature) were considered. Only studies with a minimum of 24 months of follow-up were considered. This threshold ensures that the studies provided reliable and meaningful long-term outcome data. Arthroscopic treatment for FAI involves structural modifications to the hip joint, and the recovery process and the durability of outcomes often extend beyond the early postoperative period. Studies with shorter follow-up durations may fail to capture late complications, secondary procedures, or the full extent of functional recovery, particularly in active populations such as athletes.

Future research should address the limitations identified in this systematic review. RCTs with larger sample sizes are needed to validate the findings and explore the impact of different rehabilitation protocols on RTS rates in athletes. Investigating the role of specific sport types and levels of athletic activity on surgical outcomes would provide valuable insights for personalised treatment strategies. Moreover, developing and validating sport-specific outcome measures could enhance the sensitivity of PROMs in detecting clinically meaningful changes in high-level athletes. Longitudinal studies with extended follow-up periods are essential to assess the long-term durability of hip arthroscopy outcomes and the potential for late complications, such as progression to THA.

The findings of the three studies in the present investigation indicate that hip arthroscopy is a reliable and effective treatment for FAI in both athletes and non-athletes. Although not statistically significant, the lower RTS rate among athletes suggests that they might face unique challenges in their recovery process, potentially arising from their higher baseline expectations and physical demands. These studies underscore the importance of individualised patient care and the need for tailored rehabilitation programs to address the specific needs of athletes.

## 5. Conclusions

This systematic review and meta-analysis revealed no significant differences in PROMs or complication rates between athletes and non-athletes who underwent arthroscopy for FAI at a mean follow-up of approximately two years. Both groups demonstrated substantial improvements in functional outcomes and pain relief, indicating the efficacy of hip arthroscopy across varying activity levels. Despite the limited number of included studies and some methodological biases, the findings suggest that arthroscopic intervention for FAI is equally beneficial for athletes and non-athletes. Future research should focus on long-term outcomes and tailored rehabilitation protocols to optimise recovery and return-to-sport rates.

## Figures and Tables

**Table 1:** Overview of the included studies.

Author and Year	Journal	Design	Follow-Up (Months)	Group	Patients (n)	Mean Age	Mean BMI
Ferrer-Rivero et al., 2024 [59]	J ISAKOS	Retrospective	32.4	Athletes	11	32.0	21.7
				Non-Athletes	22	32.0	21.7
Frank et al., 2019 [60]	Orthop J Sports Med	Retrospective	31.2	Athletes	97	36.0	23.8
				Non-Athletes	97	37.8	27.4
Stone et al., 2019 [61]	Arthroscopy	Retrospective	24.0	Non-Athletes	464	31.6	24.6
				Non-Athletes	60	31.6	24.6
				Athletes	47	31.6	24.6
				Athletes	10	31.6	24.6

**Table 2:** Baseline comparability (VAS: Visual Analogue Scale, HOS-ADL: Hip Outcome Score-Activities of Daily Living, HOS-SSS: Hip Outcome Score-Sport Specific Subscale). Continuous data are presented as mean value and standard deviation. Dichotomic data are reported as frequency.

Endpoint	Athletes (n = 165)	Non-Athletes (n = 643)	*p*
Mean follow-up (months)	28.8 ± 3.5	25.4 ± 2.9	0.99
Mean age	35.6 ± 1.2	32.7 ± 2.3	0.4
Mean-BMI	23.6 ± 0.6	25.1 ± 1.1	0.2
Women (%)	100% (108 of 108)	95% (556 of 583)	0.7
VAS (mean)	6.8 ± 1.8	6.7 ± 2.0	0.8
HOS-ADL (mean)	66.9 ± 16.6	55.3 ± 18.6	0.3
HOS-SSS (mean)	43.2 ± 20.3	42.1 ± 22.8	0.5

**Table 3:** Results of PROMs (VAS: Visual Analogue Scale; HOS-ADL: Hip Outcome Score-Activities of Daily Living, HOS-SSS: Hip Outcome Score-Sport Specific Subscale; MD: mean difference). Data are presented as mean value and standard deviation.

Endpoint	Athletes (n = 165)	Non-Athletes (n = 643)	MD	*p*
VAS	1.5 ± 1.9	2.1 ± 2.4	−0.5	0.7
HOS-ADL	91.6 ± 9.9	74.6 ± 24.0	17.0	0.5
HOS-SSS	82.5 ± 20.4	73.2 ± 27.6	9.3	0.4

**Table 4:** Results of endpoint rate of reoperation and progression to THA.

Endpoint	Athletes (n = 165)	Non-Athletes (n = 643)	*p*
Reoperation	3.1% (3 of 97)	2.1% (2 of 97)	0.7
Progression to THA	0% (0 of 97)	1.0% (1 of 97)	0.4

## Data Availability

Data are contained within this article or the Supplementary Materials.

## Supplementary Materials

The following supporting information can be downloaded at: https://www.mdpi.com/article/10.3390/healthcare13050470/s1, Medical subject headings (MeSH) used for database search.
