# Supplementary material for: Arthroscopy for Femoroacetabular Impingement in Athletes Versus Non-Athletes: Systematic Review and Meta-Analysis
Source: Healthcare (Basel). 2025 Feb 21;13(5):470. doi: 10.3390/healthcare13050470 (PMC11898926; doi:10.3390/healthcare13050470)
Supplement: Supplementary file 1 [file healthcare-13-00470-s001.zip › healthcare-3373522-supplementary.pdf]

Research Question:
Femoracetabular Impingement Athltes vs non-Athltes

Concept 1 – Problem: FAI

Keywords:
Acetabulum
cam
Cam impingement
cam type
FAI
FAIS
Femoroacetabular impingement
hip
Hip injury
Pincer impingement
pincer type
Sports injury

Mesh:
"Femoracetabular Impingement / complications"[Mesh]
"Femoracetabular Impingement / diagnosis"[Mesh]
"Femoracetabular Impingement / etiology"[Mesh]
"Femoracetabular Impingement / physiopathology"[Mesh]
"Fibrocartilage / physiopathology"[Mesh]
"Hip"[Mesh]
"Hip / physiopathology"[Mesh]
"Hip Joint / pathology"[Mesh]
"Pain"[Mesh]

Concept 2 - Intervention: Arthroscopy

Keywords:
arthroscopic surgical procedures
Arthroscopy
hip arthroscopic surgery
Hip arthroscopy

Mesh:
"Acetabulum / surgery"[Mesh]
"Arthralgia / surgery"[Mesh]
"Arthroscopy"[Mesh]
"Arthroscopy / methods"[Mesh]
"Cartilage, Articular / surgery"[Mesh]
"Femoracetabular Impingement / surgery"[Mesh]
"Fibrocartilage / surgery"[Mesh]
"Hip Injuries / surgery"[Mesh]
"Hip Joint / surgery"[Mesh]
"Joint Capsule / surgery"[Mesh]
"Joint Diseases / surgery"[Mesh]

Concept 3 - Comparison Athletes vs non-Athletes

Keywords:
Athletes
Athletic performance
Elite athletes
female athlete
sport type
sports

Mesh:
"Athletes"[Mesh]
"Athletic Injuries / physiopathology"[Mesh]
"Athletic Injuries / surgery"[Mesh]

Concept 4 - Outcome: PROMS

Keywords:
Athletes
Athletic performance
Elite athletes
female athlete
sport type
sports

Mesh:
"Patient Reported Outcome Measures"[Mesh]
"Patient Satisfaction"[Mesh]
"Return to Sport / statistics & numerical data"[Mesh]
"Treatment Outcome"[Mesh]
"Visual Analog Scale"[Mesh]

Searching Strategy

"Femoracetabular Impingement / complications"[Mesh] OR "Femoracetabular Impingement / diagnosis"[Mesh] OR "Femoracetabular Impingement / etiology"[Mesh] OR "Femoracetabular Impingement / physiopathology"[Mesh] OR "Fibrocartilage / physiopathology"[Mesh] OR "Hip"[Mesh] OR "Hip / physiopathology"[Mesh] OR "Hip Joint / pathology"[Mesh] OR "Pain"[Mesh] OR Acetabulum OR cam OR Cam impingement OR cam type OR FAI OR FAIS OR Femoroacetabular impingement OR hip OR Hip injury OR Pincer impingement OR pincer type OR Sports injury

AND

"Acetabulum / surgery"[Mesh] OR "Arthralgia / surgery"[Mesh] OR "Arthroscopy"[Mesh] OR "Arthroscopy / methods"[Mesh] OR "Cartilage, Articular / surgery"[Mesh] OR "Femoracetabular Impingement / surgery"[Mesh] OR "Hip Injuries / surgery"[Mesh] OR "Hip Joint / surgery"[Mesh] OR "Joint Capsule / surgery"[Mesh] OR "Joint Diseases / surgery"[Mesh] OR arthroscopic surgical procedures OR Arthroscopy OR hip arthroscopic surgery OR Hip arthroscopy

AND

"Athletes"[Mesh] OR "Athletic Injuries / physiopathology"[Mesh] OR "Athletic Injuries / surgery"[Mesh] OR Athletes OR Athletic performance OR Elite athletes OR female athlete OR sport type OR sports

AND

"Patient Reported Outcome Measures"[Mesh] OR "Patient Satisfaction"[Mesh] OR "Return to Sport / statistics & numerical data"[Mesh] OR "Treatment Outcome"[Mesh] OR "Visual Analog Scale"[Mesh] OR outcomes OR return to play OR return to sports

SUMMARY

("Femoracetabular Impingement / complications"[Mesh] OR "Femoracetabular Impingement / diagnosis"[Mesh] OR "Femoracetabular Impingement / etiology"[Mesh] OR "Femoracetabular Impingement / physiopathology"[Mesh] OR "Fibrocartilage / physiopathology"[Mesh] OR "Hip"[Mesh] OR "Hip / physiopathology"[Mesh] OR "Hip Joint / pathology"[Mesh] OR

"Pain"[Mesh] OR Acetabulum OR cam OR Cam impingement OR cam type OR FAI OR FAIS OR Femoroacetabular impingement OR hip OR Hip injury OR Pincer impingement OR pincer type OR Sports injury) AND ("Acetabulum / surgery"[Mesh] OR "Arthralgia / surgery"[Mesh] OR "Arthroscopy"[Mesh] OR "Arthroscopy / methods"[Mesh] OR "Cartilage, Articular / surgery"[Mesh] OR "Femoracetabular Impingement / surgery"[Mesh] OR "Hip Injuries / surgery"[Mesh] OR "Hip Joint / surgery"[Mesh] OR "Joint Capsule / surgery"[Mesh] OR "Joint Diseases / surgery"[Mesh] OR arthroscopic surgical procedures OR Arthroscopy OR hip arthroscopic surgery OR Hip arthroscopy) AND ("Athletes"[Mesh] OR "Athletic Injuries / physiopathology"[Mesh] OR "Athletic Injuries / surgery"[Mesh] OR Athletes OR Athletic performance OR Elite athletes OR female athlete OR sport type OR sports) AND ("Patient Reported Outcome Measures"[Mesh] OR "Patient Satisfaction"[Mesh] OR "Return to Sport / statistics & numerical data"[Mesh] OR "Treatment Outcome"[Mesh] OR "Visual Analog Scale"[Mesh] OR outcomes OR return to play OR return to sports)
